# Early insulin resistance in severe trauma without head injury as outcome predictor? A prospective, monocentric pilot study

**DOI:** 10.1186/1757-7241-20-69

**Published:** 2012-10-02

**Authors:** Manuela Bonizzoli, Giovanni Zagli, Chiara Lazzeri, Sara Degl’Innocenti, Gianfranco Gensini, Adriano Peris

**Affiliations:** 1 Anaesthesia and Intensive Care Unit of Emergency Department, Careggi Teaching Hospital, Largo Brambilla 3, 50139 Florence, Italy; 2 Heart and Vessel Department, Careggi Teaching Hospital, Largo Brambilla 3, 50139, Florence, Italy

**Keywords:** Trauma, Insulin resistance, Outcome, Intensive care unit

## Abstract

**Background:**

Hyperglycemia following major trauma is a well know phenomenon related to stress-induced systemic reaction. Reports on glucose level management in patients with head trauma have been published, but the development of insulin resistance in trauma patients without head injury has not been extensively studied. The aim of this study was therefore to investigate the prognostic role of acute insulin-resistance, assessed by the HOMA model, in patients with severe trauma without head injury.

**Methods:**

All patients consecutively admitted to the Intensive Care Unit (ICU) of a tertiary referral center (Careggi Teaching Hospital, Florence, IT) for major trauma without head injury (Jan-Dec 2010) were enrolled. Patients with a previous diagnosis of diabetes mellitus requiring insulin therapy or metabolism alteration were excluded from the analysis. Patients were divided into “insulin resistant” and “non-insulin resistant” based on the Homeostasis Model Assessment index (HOMA IR). Results are expressed as medians.

**Results:**

Out of 175 trauma patients admitted to the ICU during the study period, a total of 54 patients without head trauma were considered for the study, 37 of whom met the inclusion criteria. In total, 23 patients (62.2%) resulted insulin resistant, whereas 14 patients (37.8%) were non-insulin resistant. Groups were comparable in demographic, clinical/laboratory characteristics, and severity of injury. Insulin resistant patients had a significantly higher BMI (P=0.0416), C-reactive protein (P=0.0265), and leukocytes count (0.0301), compared to non-insulin resistant patients. Also ICU length of stay was longer in insulin resistant patients (P=0.0381).

**Conclusions:**

Our data suggest that admission insulin resistance might be used as an early outcome predictor.

## Introduction

Hyperglycemia following major trauma is a well know phenomenon related to stress-induced systemic reaction [1]. Despite the recognized importance of glucose control, in major trauma with head injury, strict glucose control might be not appropriate since the risk of hypoglycemia (even transient) is as dangerous as uncontrolled hyperglycemia for the injured brain [2]. In this context, studies available cannot clarify if early hyperglycemia, such as epiphenomena of stress-induced insulin resistance, can be considered an outcome parameter in politrauma without head injury, in which strict glucose control can be done more properly. Little data is available on the prognostic role of acute insulin-resistance in trauma patients in absence of head injury.

The aim of this study was therefore to investigate the prognostic role of acute insulin-resistance, as assessed by the Homeostatic Model Assessment (HOMA index) in patients with severe trauma without head injury.

## Methods

### Patient selection and study design

This is a prospective, pilot study in which all patients consecutively admitted to the ICU of a tertiary referral center (Careggi Teaching Hospital, Florence, IT) for major trauma without head injury from January to December 2010 were enrolled. For each patient, data from ICU databases and the Italian Group for the Evaluation of Interventions in Intensive Care Medicine database (GiViTI Margherita Project, Istituto Mario Negri, Bergamo, Italy) were collected: age, gender, body mass index (BMI), medical history, injury severity score (ISS), simplified acute physiology score (SAPS) II, Sequential Organ Failure Assessment (SOFA score), duration of mechanical ventilation, ICU length of stay (LOS).

Past medical history was obtained through patient interviews or from the families of those unable to be interviewed. Patients with a previous diagnosis of diabetes mellitus requiring insulin therapy or alteration in fat acid metabolism were excluded from the analysis.

The Internal Review Board approved of the study, and informed consent for study participation and data publication was obtained from patients.

### Definition of insulin resistance and outcome parameters

Criteria used for the definition of insulin resistance are in accordance with the recently published guidelines proposed by the European Group of the study of Insulin Resistance (EGIR: http://www.egir.org). Patients were divided into “insulin resistant” and “non-insulin resistant” groups, based on the Homeostasis Model Assessment – HOMA, calculated for each individual. Subjects whose values exceeded the sex-specific 75^th^ percentile (i.e. 1.80 for women and 2.12 for men) were considered to have insulin resistance (Homeostasis Model Assessment - HOMA-IR) [3][4][5].

As primary outcome end points we considered ICU mortality and ICU LOS. Secondary end points were days of mechanical ventilation and infection (sum of pneumoniae and wound infection) rate.

### Statistical analysis

Statistical analyses were carried out by SPSS 18 (SPSS Inc., Chicago, Illinois). Continuous variables were analysed with two-tail Student's *t*-test or Mann–Whitney test, as appropriate (D'Agostino & Pearson normality test). Continuous variables are expressed as medians with 25^th^ to 75^th^ interquartile range (IQR). Categorical variables were examined using Fisher’s exact test. A P value below 0.05 was considered an index of statistical significance. Univariable comparison was reported as odds ratio (OR) with 95% confidence intervals (CI).

A logistic regression model was adopted to investigate the predictors of insulin resistance index in overall population. Each predictor likely related to the outcome was evaluated according to statistical and clinical bases. Covariates associated with the response variables (p≤0.2) in univariate analysis were retained in the multivariate model (P<0.05).

## Results

Out of 175 trauma patients admitted to the ICU during the whole study period, a total of 54 patients without head trauma were considered for the study, 37 of whom met the inclusion criteria and were therefore enrolled. Demographics and clinical characteristics are shown in Table 1. According to HOMA index positivity, 23 patients were classified as “insulin resistant”, whereas 14 patients were included in the non-insulin resistant group. Groups resulted comparable in demographic characteristics and severity of trauma (ISS score), however, they significantly differed in BMI (25.7 vs 23.5 Kg/m^2^, p=0.0416), and ICU LOS (8 vs 4.5 days, p=0.0381) (Table 1).

**Table 1:** **Comparison of baseline and clinical characteristics in overall population, insulin resistant patients, and non**-**insulin resistant patients**

	**Overall**	**Insulin resistant patients**	**Non**-**insulin resistant patients**	**P**
**Number**,% (**N**)	37	62.2% (23)	37.8% (14)	
**Age** (**years**)	38.5 (29.8-60.3)	41.5 (32.8-62.8)	37.5 (27.5-54.3)	0.3142
**Male sex**, **N** (%)	75.7% (28)	73.9% (17)	78.6% (11)	1.0000
**BMI** (**Kg**/**m**^**2**^)	24.7 (22.8-26.1)	25.7 (22.7-26.2)	23.5 (22.9-24.6)	**0**.**0416**
**SAPS II**	31 (19–45)	33 (21.5-48)	19.5 (17–39.8)	0.1322
**ISS**	29 (22–36)	27 (20.5-36)	29 (27–36)	0.5609
**SOFA score**	6 (3–9)	7 (4.5-10.5)	4 (3–7.5)	0.0957
**Infections during ICU stay**,% (**N**)	46% (17)	52% (12)	36% (5)	1.0000
**Duration of mechanical ventilation** (**days**)	4 (1–11)	7 (1–12)	2 (1–5)	0.2887
**LOS ICU** (**days**)	7 (3–16)	8 (4–15)	4.5 (3–14)	**0**.**0381**
**ICU mortality**,% (**N**)	5% (2)	9% (2)	0	1.0000

Continuous data are analysed with two-tail Student's *t*-test or Mann–Whitney test, as appropriate (D'Agostino & Pearson normality test). Continuous variables are expressed as medians with 25^th^ to 75^th^ interquartile range (IQR). Categorical variables were examined using Fisher’s exact test. P significant if <0.05 (bold).

*AIS* Abbreviated Injury Scale, *BMI* Body Mass Index *ISS* Injury Severity Score, *ICU* Intensive Care Unit, *LOS* Length Of Stay, *SAPS* Simplified Acute Physiology Score, *SOFA* Sequential Organ Failure Assessment.

Table 2 shows laboratory parameters upon ICU admission. Insulin-resistant patients resulted as having a significantly higher C-reactive protein (140.5 vs 83, p=0.0265, respectively) and leukocytes count (11,100 vs 8,200 cells/ml, p=0.0301, respectively) than non-insulin resistant patients. Also, C-peptides were significantly higher in insulin resistant patients (0.95 vs 0.43, p=0.0061), whereas glycated hemoglobin resulted comparable between the groups (Table 2). The subsequent logistic regression model did not show significant correlation among variables and outcome parameters.

**Table 2:** **Comparison of laboratory data at ICU admission in overall population, insulin resistant patients, and non**-**insulin resistant patients**

	**Overall**	**Insulin resistant patients**	**Non**-**insulin resistant patients**	**P**
**Number**,% (**N**)	37	62.2% (23)	37.8% (14)	
**Leukocytes** (n*1000/**ml**)	9.6 (7.9-13.1)	11.1 (9.1-3.7)	8.2 (7.8-9.5)	**0**.**0301**
**Haemoglobin** (**gr**/**dl**)	10.1 (9–11.4)	9.8 (9.2-11)	10.1 (9–11.9)	0.7441
**Fibrinogen** (**mg**/**dl**)	361 (317–525)	359 (313–527)	375 (345–490)	0.8413
**Antithrombin III** (%)	74 (64–87)	71 (65–86)	79 (62–88)	0.6885
**d**-**dimers** (μ**g**/**l**)	3455 (2034–5960)	3655 (2591–5415)	3198 (1962–6387)	0.7398
**Blood glucose** (**mmol**/**l**)	7 (6.1-7.4)	7.2 (6.5-7.4)	6.3 (5.7-7.4)	0.1578
**Plasma insulin** (**mU**/**l**)	9.90 (4.78-19.48)	15.55 (11.10-24.08)	4.55 (3.23-5.48)	<**0**.**0001**
**HOMA index**	3.20 (1.46-5.76)	4.72 (3.65-8.84)	1.27 (0.983-1.57)	<**0**.**0001**
**C**-**peptides** (**mmol**/**l**)	0.72 (0.46-1.05)	0.95 (0.53-1.12)	0.43 (0.32-0.65)	**0**.**0061**
**Glycated hemoglobin**,%	5.4 (5.2-5.6)	5.5 (5.3-5.7)	5.4 (5.2-5.6)	0.2067
**Total plasma proteins** (**g**/**dl**)	4.80 (4.20-5.20)	4.7 (4.3-5.2)	4.8 (4.3-5.2)	0.9875
**Plasma albumin** (**g**/**l**)	25.70 (20.35-30.45)	25.7 (19.3-29.4)	26.1 (23.7-31.3)	0.4042
**Arterial lactates** (**mmol**/**l**)	1.15 (0.80-2)	1.2 (0.9-1.9)	1.3 (0.8-2)	0.8754
**C**-**reactive protein** (**mg**/**l**)	114 (74–165.5)	140.5 (90.8-197.8)	83 (70–97)	**0**.**0265**
**Triglycerides** (**mg**/**dl**)	77 (53–114)	88 (64–150)	57 (46–88)	0.2351
**Total cholesterol** (**mg**/**dl**)	106 (82–112)	108 (104–123)	83 (75–109)	0.0700
**Cholesterol HDL** (**mg**/**dl**)	26 (22–33)	26 (25–34)	24 (18–31)	0.2635
**AST** (**U**/**l**)	72 (35–165)	63 (38–169)	68 (34–155)	0.9388
**ALT** (**U**/**l**)	43 (28–122)	40 (28–69)	48 (30–154)	0.4659
**CPK** (**U**/**l**)	1556 (891–3531)	1336 (696–4228)	2040 (1149–3215)	0.5702
**LDH** (**U**/**l**)	537 (240–500)	297 (237–495)	391 (345–507)	0.1670
**Myoglobin** (**ng**/**ml**)	993 (340–1824)	762 (351–2475)	1316 (314–1574)	0.3185

Continuous data are analysed with two-tail Student's *t*-test or Mann–Whitney test, as appropriate (D'Agostino & Pearson normality test). Continuous variables are expressed as medians with 25^th^ to 75^th^ interquartile range (IQR). P significant if <0.05 (bold).

*ALT* alanine aminotransferase, *AST* aspartate aminotransferase, *CPK* creatine phosphokinase, *HOMA* Homeostasis Model Assessment, *LDH* lactate dehydrogenase, *NT-pro-BNP* N-terminal-pro-brain natriuretic peptide.

## Discussion

The present investigation, to our knowledge, represents the first study concerning the prognostic role of admission insulin resistance in trauma patients as a potential tool for a better early risk stratification in these patients.

The main finding in 37 patients with major trauma without head injury and without previously known diabetes is that admission acute insulin resistance (as assessed by means of the HOMA model) is a common finding, and it is associated with a longer ICU stay. Blood glucose levels were similar between the groups thanks to the higher insulin secretion in insulin-resistant patients: this phenomenon, in our opinion, merits further analysing since the role of glucose control in critically ill patients is usually considered only in the case of normal glycaemia. Our data, despite all limitations, suggest that even higher insulin secretion can have a role in organ dysfunction following major trauma.

Insulin resistance was significantly associated with a longer ICU LOS (8 vs 4.5 days, p=0.0381; Table 1), but we failed to verify the correlation with mortality, ventilation days, and infection rate. Moreover, insulin resistant patients differed (without reaching statistical significance) from non-insulin resistant patients also in SAPS II score (33 vs 19.5, respectively), SOFA score (7 vs 4, respectively), infections during ICU stay (52% vs 36%, respectively), and duration of mechanical ventilation (7 vs 2 days, respectively) (Table 1). The lack of statistical significance might be attributed to the limited sample size of the study, which can be considered the main limitation of our work. Other important limitation factors are the non-randomized and monocentric design of the study. Nevertheless, despite these limitations, the possibility that admission HOMA-IR could be a predictive factor in major trauma without head injury is very intriguing.

Despite many studies on hyperglycaemia, very little data is available on acute insulin resistance and its effect on trauma patients’ outcome. Laird and co-authors [6] showed in 2004 that hyperglycemia (defined as glucose level >200 mg/dl, or >11 mmol/l) in trauma patients at admission was associated with significantly higher infection and mortality rates. Even a mild admission hyperglycemia (>150 mg/dl, or >8.3 mmol/l) was recognised as an independent predictor for mortality, postoperative infection, and hospital and Intensive Care Unit (ICU) length of stay [7][8]. Bochicchio and co-workers reported that critically ill trauma patients with a persistent high glucose level (>140 mg/dl, or >7.8 mmol/l) had a statistically significant ICU and hospital length of stay, as well as mortality, even after adjusting for age and Injury Severity Score (ISS) [9]. Yendamuri and co-authors showed in 2003 that even mild hyperglycemia (>7.5 mmol/l) was a prognostic factor in an overall trauma population, and not only in brain injured patients [7]. More recently, Mowery and co-authors analysed insulin resistance in a wide range of traumatic brain injured patients finding that insulin resistance was a prognostic factor independent from strict glucose control [10], but the opportunity to perform strict glucose control in traumatic brain injured patients remains questionable, since the effect of hypoglycemic episodes are considered as dangerous as hyperglycemia [2]. Despite numerous studies on glucose metabolism in traumatic brain injured patients or in overall trauma populations, as mentioned, very little data is available on insulin resistance in major trauma patients without brain injury. In our sample, the fact that hyperglycemia (secondary to insulin resistance) might have significance as a prognostic factor could make it possible to easily perform the appropriate intervention, since the absence of brain injury permits more aggressive glucose control.

In our study population, stress reaction following major trauma without head injury caused secondary insulin resistance in 62.2% of patients. The definition of “secondary” came from the inclusion criteria (patients with history of glucose metabolism alteration were excluded) and from the confirmation of glycated hemoglobin dosage, which resulted as comparable between groups (Table 2). The development of insulin resistance can be related to post-traumatic systemic inflammation, even considering the higher leukocytes count and C-reactive protein dosage in insulin resistant patients. Our finding is in agreement with data reported in experimental rat models, in which it has been observed that trauma alone results in modest insulin resistance, occurring gradually [11,12]. However, when injury is combined with haemorrhage, a disorder in glucose metabolism rapidly develops, occurring in rat liver as soon as 15 min following trauma and haemorrhage. This is characterized by severe defects in hepatic insulin signalling, hyperinsulinemia and hyperglycemia [12-16].

## Conclusions

In the present single-centre investigation, we found that a large proportion of patients with major trauma but no (or without) head injury developed insulin resistance after accident as a result of systemic stress reaction. It might be interesting to be reproducing this phenomenon, associated with a longer ICU stay in our population, in other centres and in larger populations, with the aim to understand if early insulin-resistance could be included in prognostic scoring.

## Key messages

• Acute insulin resistance has been found to be common in severe trauma without head injury and without glucose metabolism alterations.

• Insulin resistance seems to be linked with acute stress reaction.

• The develop of insulin resistance results to be a predictor of ICU length of stay.

## Abbreviations

BMI: Body Mass Index; HOMA: Homeostatic Model Assessment; ICU: Intensive Care Unit; ISS: Injury Severity Score, LOS: Length Of Stay; SAPS: Simplified Acute Physiology Score; SOFA: Sequential Organ Failure Assessment.

## Competing interests

The authors declare that they have no competing interests (financial or non-financial).

## Authors’ contributions

MB, CL, GGF, and AP designed the study; MB, CL, and GZ reviewed the literature; SDI collected data; GZ performed statistical analysis; GZ, CL, MB and AP wrote draft. All authors revised and approved the manuscript.
